# Status of birth and pregnancy outcome capture in Health Demographic Surveillance Sites in 13 countries

**DOI:** 10.1007/s00038-019-01241-0

**Published:** 2019-06-26

**Authors:** Peter Waiswa, Joseph Akuze, Cheryl Moyer, Doris Kwesiga, Samuelina Arthur, Osman Sankoh, Paul Welaga, Martin Bangha, Jacques Eminas, Sheru Muuo, Abdhalah Ziraba, Kate Kerber, INDEPTH Network MNCH team, Estelle Mclean, Sulaimon Afolabi, Rhian Twine, Pallavi Lele, Sanjay Juvekar, Muluemebet Abera, Fasil Tessema, David Obor, Jennifer Verani, Dan Kajungu, Edward Galiwango, Seni Kouanda, Adama Baguiya, Peter Sifuna, Walter Otieno, J. Anthony G. Scott, Mark Otiende, Margaret May, Jessica Price, Donatien Beguy, Nega Assefa, Siaka Kone, Juerg Utzinger, Alemseged Aregay Gebru, Loko Abraham, Shashi Kant, Partha Haldar, Ane Fisker, Amabelia Rodrigues, Gashaw Andargie, Kassahun Alemu, Robert Newton, Gershim Asiki, Margaret Gyapong, Vida Kukula, Halidou Tinto, Karim Derra, Daniel Azongo, Wubegzier Mekonen, Mitike Molla, Abdramane Bassiahi Soura, Souleymane Sanog, Dorean Nabukalu, Tom Lutalo, Yeetey Enuameh, Alexander Manu, Obed Ernest Nettey, Abdul Wahab, Siswanto Agus Wilopo, Anne Rerimoi, Momodou Jasseh, Mamadou Ouattara, Eric Diboulo, Morris Ndemwa Mwangangi

**Affiliations:** 1 INDEPTH Network Maternal Newborn and Child Health Working Group, Kampala, Uganda; 2 Department of Health Policy Planning and Management, Karolinska Institutet, Kampala, Uganda; 3 University of Michigan, Ann Arbor, USA; 4 Department of Epidemiology and Population Health, London School of Hygiene and Tropical Medicine, London, UK; 5 Statistics Sierra Leone, Tower Hill, Freetown, Sierra Leone; 6 College of Medicine and Allied Health Sciences, University of Sierra Leone, Freetown, Sierra Leone; 7 School of Public Health, Faculty of Health Sciences, University of the Witwatersrand, Johannesburg, South Africa; 8 University of Alberta, Edmonton, Canada; 9 African Population and Health Research Centre, Nairobi, Kenya; 10 Navrongo Health Research Centre, Navrongo, Ghana; 11 Global Health Division, Karolinska Institutet, Solna, Sweden

**Keywords:** INDEPTH Network, Maternal Newborn Child Health Working Group, Stillbirth, Neonatal, Mortality, Perinatal mortality, Demographic Surveillance Sites

## Abstract

**Objectives:**

We compared pregnancy identification methods and outcome capture across 31 Health Demographic Surveillance System (HDSS) sites in 14 countries in sub-Saharan Africa and Asia.

**Methods:**

From 2009 to 2014, details on the sites and surveillance systems including frequency of update rounds, characteristics of enumerators and interviewers, acceptable respondents were collected and compared across sites.

**Results:**

The 31 HDSS had a combined population of over 2,905,602 with 165,820 births for the period. Stillbirth rate ranged from 1.9 to 42.6 deaths per 1000 total births and the neonatal mortality rate from 2.6 to 41.6 per 1000 live births. Three quarters (75.3%) of recorded neonatal deaths occurred in the first week of life. The proportion of infant deaths that occurred in the neonatal period ranged from 8 to 83%, with a median of 53%. Sites that registered pregnancies upon locating a live baby in the routine household surveillance round had lower recorded mortality rates.

**Conclusions:**

Increased attention and standardization of pregnancy surveillance and the time of birth will improve data collection and provide platforms for evaluations and availability of data for decision-making with implications for national planning.

**Electronic supplementary material:**

The online version of this article (10.1007/s00038-019-01241-0) contains supplementary material, which is available to authorized users.

## Introduction

At the end of the Millennium Development Goals (MDGs) era, the number of children dying each year before the age of five dropped by 54%, from an estimated 12.7 million in 1990 to fewer than six million in 2015. In addition, a 44% reduction in maternal mortality ratio (MMR), from 385 per 100,000 live births in 1990, to 216 per 100,000 live births in 2015 was seen. Despite great progress in reducing child deaths during the MDGs, neonatal mortality had the least progress and now accounts for 44% of these deaths globally (UNICEF et al. 2015). An estimated 2.6 million newborns die each year (IGME 2017), whereas 2.6 million babies stillborn (Blencowe et al. 2016). Furthermore, the burden of disability and ill health arising out of the neonatal period is substantial. Neonatal conditions accounted for 202 million disability-adjusted life years in 2010, or 8.1% of the worldwide total (Murray et al. 2012). The risk of death is highest during the birth process and the days immediately after, across all countries and income levels. Approximately 36% of neonatal deaths occur on the first day and 73% in the first week after birth (Oza et al. 2014). The progress in stillbirths was even less, with an estimated decline from 24.7 stillbirths per 1000 total births in 1990 to 18.4 stillbirths per 1000 total births in 2015, a 2% annual reduction rate (de Bernis et al. 2016; Lawn et al. 2016).

The Sustainable Development Goals (SDGs) which were launched in 2016 set ambitious targets to be achieved by 2030. Goal 3 of the SDGs focuses on health, and its vision is “to ensure healthy lives and promote wellbeing for all at all ages”. The SDGs target a MMR of less than 70 deaths per 100,000 live births globally or not more than 140 deaths per 100,000 live births for any county by 2030. The under-five mortality target is 25 deaths per 1000 live births by 2030 (UN 2015). The targets set in the SDGs and their accompanying strategies are ambitious; however, to achieve them we require improved, timely metrics, but also innovation and policies based on local evidence, especially in low- and middle-income countries. Mortality must be measured in order to guide the achievement of the Every Newborn Action Plan (ENAP) vision to end preventable newborn mortality and stillbirths, to determine whether the goals have been met, to monitor coverage of interventions and to ensure rapid feedback to evaluate whether the interventions are reaching those in need, especially populations at risk. To this end, the 5-year multi-partner ENAP Measurement Improvement Roadmap details steps to meet the key ENAP milestones, including better coverage and quality of birth and death information, with improvements in data collection tools for measuring mortality outcomes like neonatal mortality rates (NMR) and stillbirth rates (SBR) (Moxon et al. 2015).

Despite this increasing attention to stillbirths (Froen et al. 2016) and neonatal deaths(Shiffman 2010), little longitudinal information is gathered on these deaths and their surrounding circumstances in low- and middle-income countries (LMIC). The burden of stillbirths and neonatal deaths is greatest where local data for planning and action are neither readily available nor accurate. Few large-scale studies that document perinatal outcomes exist in a community setting (Engmann et al. 2012).

The International Network for the Demographic Evaluation of Populations and their Health (INDEPTH) operates in these high-burden settings, through an affiliated group of Health and Demographic Surveillance Systems (HDSS) sites. These sites monitor demographic, social, health and other characteristics of target populations. Founded in 1998, by December 2015 it had brought together 49 HDSS field sites from 43 member research centres in 20 countries (INDEPTH Network). Member sites collect data on various indicators (Sankoh and Byass 2012).

The ongoing surveillance in INDEPTH sites makes them valuable resources for improving the understanding of birth outcomes in the LMICs. Their longitudinal nature positions them as a great platform for assessing the lifecycle changes from pregnancy until death. We examined available data and compared pregnancy identification and outcome capture across INDEPTH Network HDSS sites in 13 countries in sub-Saharan Africa and Asia. By doing so, we aim to contribute to improving methods for identifying pregnancies and capturing their outcomes at the population level, a key ENAP requirement.

## Methods

### Data collection and management

All INDEPTH member sites were invited to participate in the INDEPTH Network Maternal, Newborn and Child Health Working Group (MNCH_WG) activities within the INDEPTH Network because the network membership criteria require all sites to collect data on pregnancy and their outcomes and also to conduct maternal and newborn causes of deaths using verbal autopsy. However, participation is not mandatory because it depends on HDSS sites’ interests and the nature of their core research activities. The data for this analysis were solicited from all INDEPTH Network member HDSS sites using a standard template sent out to teams to complete. This request was sent by the INDEPTH Network secretariat hosted in Accra, Ghana, to all the 48 INDEPTH Network member sites at the time and not only to the MNCH_WG member sites. Of 48 sites contacted, 31 responded and returned the data request template to the secretariat. One site had not yet been added to the INDEPTH membership at the time of data collection. Sites were asked to submit 2009–2014 data on the crude population numbers; number of pregnancies; the pregnancy outcomes including live birth, stillbirth, neonatal death and timing of death; details of the birth, including gestational age, delivery location; and background characteristics, including age of mother, mother’s education, household wealth index. However, majority of the sites did not provide the detailed data on the births and the background characteristics of the mother; therefore, these data are not presented in this paper for consistency. The data that the sites provided were collected by each HDSS as a part of their routine monitoring and surveillance. Because of data ownership issues, a protocol was sent to the HDSS site teams to guide them to analysing their own datasets and enter the data in a standardized, structured Excel forms which were then shared with the research team via webmail. The outcome definitions used in this paper are detailed in Table 1.

**Table 1:** Outcome definitions. *Source*: Moxon et al. (2015) (9)

Indicator	Numerator	Denominator
Stillbirth rate	International comparison definition: number of babies born per year with no signs of life weighing at least 1000 g and after 27 completed weeks of gestation or more than 35 cm ICD-10 definition: number of babies born per year with no signs of life weighing at least 500 g and ≥ 22 weeks completed weeks of gestation	1000 total (live and stillborn) births
Neonatal mortality rate	Number of live born infants per year dying before 28 days of life (0–27 days)	1000 live births
Early neonatal mortality rate	Number of live born infants per year dying before 7 days of life (0–6 days)	1000 live births
Infant mortality rate	Number of infants per year dying before 12 months of life (365 days)	1000 live births

Definitions of outcomes used in the study 2009–2014. ICD-10: International Classification of Diseases version 10; CRVS: civil registration and vital statistics; HMIS: health management information system

The details on the HDSS context and the characteristics of the surveillance rounds including the frequency of surveillance updates, the characteristics of interviewers and acceptable respondents were also collected through a review of the HDSS profiles and published manuscripts on the INDEPTH Network website; we collated these and compared across sites. All data received via the Excel templates and the reviews on the HDSS profiles were compiled and analysed by the lead author team. Site representatives and maternal newborn focal persons were sent the draft manuscript via email to review their data that were compiled by the author team; this was followed up with a meeting and discussions about the data during workshops in Kampala in June 2015 and in Addis Ababa in November 2015. After the face-to-face meetings and discussions, the HDSS site were sent updates and revisions made in June 2016.

### Data analysis

The data were analysed using Stata/SE 14 and MS Excel software. Descriptive analyses were conducted to describe the birth and mortality profile across sites.

Stillbirth rates, early, late and overall NMR and infant mortality rates were calculated using the raw data provided by the sites and averaged over the 5-year data available. They were compared to national and regional estimates available from other sources including global estimates and large-scale household surveys. The ratio of stillbirths to neonatal deaths and the proportion of infant deaths that occurred during the neonatal period were calculated, and we compared the stillbirth rates and NMR to national and global averages. The stillbirth rates, early and overall NMR and infant mortality rates of seven sites were excluded or not shown because either they did not provide data on neonatal deaths or the neonatal mortality rates computed were less than 10 deaths per 1000 live births—a cut point that we set for what could be considered realistic mortality rates given the low-resource settings of INDEPTH HDSS.

## Results

Out of the 48 sites that were contacted, 31 sites replied (65% response rate). Data from the 17 sites that did not respond are not included in this analysis, but characteristics of all 48 sites are included in Supplementary table 2. The 31 sites that submitted data were located in thirteen countries, representing three major regions (Central and South Asia, sub-Saharan Africa and Eastern and South-eastern Asia). Although data for the period of interest were from the years 2009 to 2014, the latest data for the 31 sites submitted varied from 2010 to 2014, with 3% from 2010, 32% from 2011, 26% from 2012, 16% from 2013 and 23% from 2014. The 31 sites had a combined population of over 2.9 million, with 165,720 births recorded in 2014 for the period 2009–2014. Their population ranged from 20,630 to 268,783 and their size from 135 to 7162 square kilometres.

### Stillbirths

**Fig. 1:**
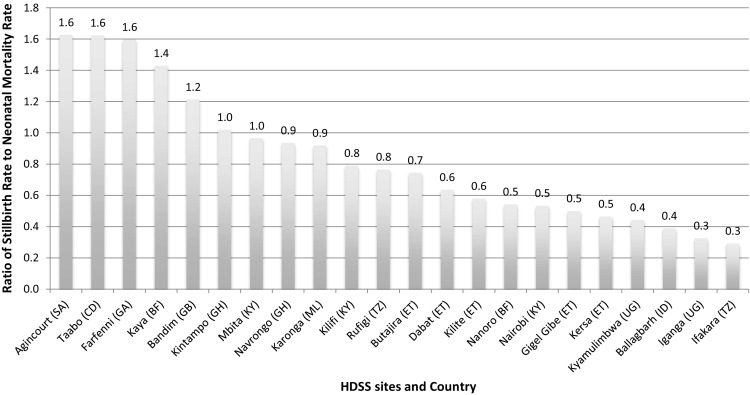
Ratio of stillbirth rate to neonatal mortality rate across sites. Ratio of stillbirth rate to neonatal mortality rate from 22 Health and Demographic Surveillance Sites for the period 2009–2014 [Key: (SA)—South Africa; (CD)—Côte d’Ivoire; (GA)— Gambia; (BF)—Burkina Faso; (GB)—Guinea-Bissau; (KY)—Kenya; (GH)—Ghana; (ML)—Malawi; (TZ)—Tanzania; (ET)—Ethiopia; (UG)—Uganda; (ID)—India]

The sites had differential capacity in measuring different rates. Stillbirth rates ranged from 7 deaths per 1000 total births, to 42.6 deaths per 1000 total births, with a median stillbirth rate of 17 per 1000 total births across sites (Supplementary table 3). The ratio of SBR to NMR ranged from 0.3 to 1.6, with a median ratio of 0.75 (Fig. 1 and Supplementary table 3). Most sites only collected the most basic information on stillbirths and did not collect data on whether or not the stillbirth occurred in the intrapartum or antepartum period or appeared fresh or macerated at delivery. Out of 24 sites presented in Supplementary table 3, 23 sites had data on stillbirths. Of the 23 sites with stillbirth data, 19 HDSS sites had a 5-year average stillbirth rate that was lower than national estimates for 2015 (Supplementary table 3).

### Neonatal deaths

**Fig. 2:**
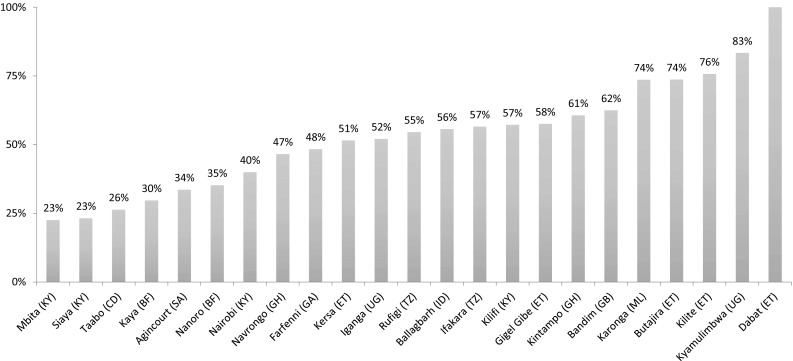
Proportion of infant deaths (first year) that occur in the neonatal period (first month) across sites. Proportion of infant deaths that are neonatal deaths from 22 Health and Demographic Surveillance Sites for the period 2009–2014 [Key: (SA)—South Africa; (CD)—Côte d’Ivoire; (GA)— Gambia; (BF)—Burkina Faso; (GB)—Guinea-Bissau; (KY)—Kenya; (GH)—Ghana; (ML)—Malawi; (TZ)—Tanzania; (ET)—Ethiopia; (UG)—Uganda; (ID)—India]

Among the 31 sites, 28 submitted data on the number of neonatal deaths and the age of the newborn at the time of death. Of these 28 sites, four sites had an NMR that was less than 10 deaths per 1000 live births. These unrealistically low rates were excluded. The NMR in the remaining 24 sites ranged from 11 deaths per 1000 live births to 41.6 deaths per 1000 live births, with a median NMR of 21.4 across sites and a median early NMR (a death in the first 7 days after birth) of 14.2 (Supplementary table 3). The proportion of all neonatal deaths that occurred during the first week was 71.4% across sites. The proportion of infant deaths (first year deaths) that occurred during the neonatal period ranged from 22.6 to 100%, with a median of 55.1% across sites (Fig. 2 and Supplementary table 2). Out of 24 sites with data, neonatal deaths or neonatal mortality greater than 10 deaths per 1000 births, 15 HDSS sites had a 5-year average NMR which were lower than national estimates of NMR for 2014 (Supplementary table 3).

### Surveillance characteristics

Table 2 highlights surveillance processes in sites. Fifteen sites had more frequent surveillance rounds, ranging from monthly to quarterly rounds. The other sixteen sites with infrequent rounds conducted them either once or twice a year. The majority of the sites collect data relevant to birth outcomes including maternity history, pregnancy and outcome surveillance, child health, verbal autopsies, reproductive health, immunization, among others. Seven sites asked questions directly to women of childbearing age in the household, while 15 asked any adult respondent present in the home, not necessarily a woman. Seventeen sites registered pregnancies upon locating a live baby in the routine household surveillance round or through community notifiers. Of the 31 sites, 18 collected data using a paper-based system and this was then entered into a database, while 8 used electronics-based data collection. Most of the sites stored their entered data in either an SQL or DBASE or Open Household Registry systems.

**Table 2:** Characteristics of surveillance systems

HDSS name	Country	Frequency of rounds	Information collected relevant to birth outcomes	Data collector	Acceptable respondent	Other information
Africa Centre	South Africa	Three update rounds a year	All births, deaths, marriages and migrations, socio-economics, biometrics, HIV status and sexual behaviour		Senior household member	The data are stored in a single Microsoft SQL server database
Agincourt	South Africa	One annual round	Migration, death, maternal history, pregnancy and outcome surveillance	Four teams, one supervisor, eight fieldworkers	Household head, spouse or adult member in the house	The database is managed using Microsoft SQL servers
Ballabgarh	India	(Monthly) Pregnancy, gestational age, stillbirth outcomes and birthweight	Migration, antenatal care, immunization, family planning, morbidity and verbal autopsy			The database is handled in DBASE III and Microsoft SQL
Bandim	Guinea-Bissau	Monthly in the urban site and biannually in the rural site	Vaccination and vitamin A supplementation	Fieldworkers		
Butajira	Ethiopia	(Quarterly) pregnancy surveillance: each household has been visited once per month from 1987 to 1999 and four times in year since 1999	Child health, mortality patterns, health systems and financing, verbal autopsy, malaria, nutrition status and domestic violence	21 Data collectors and 7 supervisors, 2 field coordinators	Household head/spouse and adult member of the family	The data are entered into the Household Registration System 2 (HRS2) and stored on the FoxPro server
Dabat	Ethiopia	(Biannually) pregnancy, gestational age, delivery and stillbirth outcomes	Births, verbal autopsy, migrations, pregnancies and pregnancy outcomes		Adult women or Mothers of the households	Data are collected on paper and entered using Dabat database Household Registration System 2
Dodowa	Ghana	(Biannually) pregnancy, delivery, stillbirth outcomes, birthweight	Verbal autopsy, migration and pregnancy, spatial data and outcome surveillance			The data are entered into the Household Registration System 2 and store on the Microsoft SQL server
Farfenni	Gambia	(Quarterly) pregnancy, birthweight and neonatal morbidity	Verbal autopsy, vaccination, migration, pregnancy registration and outcome and spatial data			The data are entered into the Household Registration System 2 and store on the Microsoft SQL server
Gilgel Gibe	Ethiopia	Biannual population update, pregnancy outcome, death, migration, martial change among women, pregnancy observation. Yearly updates for education, marital status and occupation	Maternal and child health, verbal autopsy	Men and women who completed at least secondary education	Household head, spouse or adult member in the house	Household Registration System 2 database is used for data entry. Data are collection is paper-based and planned to be migrated to OpenHDSS. The HDSS site is owned and run by Jimma University
Ifakara	Tanzania	Three update rounds a year. With four months for each round starting at January, May and September	Pregnancies, births deaths			
Iganga-Mayuge	Uganda	Two update rounds per year with a round lasting about 2-3 months	Births, deaths, migrations, pregnancy registration and outcome surveillance, vaccination, socio-economic characteristics and verbal autopsy	Research assistants, scouts, Village Health Teams (VHT) members	Adult member of the household	The data are collected using paper-based system and is entered into the Household Registration System 2 and stored on the MySQL server. The site will migrate to OpenHDSS in 2017
Karonga	Malawi	Births and deaths are reported monthly and in-and-out migration is collected annually	Migration, vaccination, verbal autopsy	Fieldworkers	Local “key informants” who may not be a member of that household or household member	Data are collected on paper and I entered into a MS Access database
Kaya	Burkina Faso	(Biannually) pregnancy and stillbirth outcomes	Births, verbal autopsy, marriage, migration, pregnancies and morbidity		Member of the household	Data are collected using personal digital assistants using the CSPro software and is saved on the MySQL–PHP database
Kersa	Ethiopia	(Quarterly) Pregnancy, gestational age, delivery, stillbirth outcomes, birthweight information, neonatal and under-five morbidity surveillance	Maternal and child health and verbal autopsy		Preferably head of the household, mother, if not children in the house who are older than 15 years	Data are collected using paper, consistency and completeness check is done by resident supervisors and field coordinators. Once completed entered in HRS 2 software and Hardcopy archived
Kilifi	Kenya	(Three times per year) pregnancy, delivery, stillbirth outcomes, maternal outcomes	Migration, verbal autopsy, births, pregnancy surveillance and vaccination			The data are managed in a central server database which is specified in FileMaker Pro version 11
Kilte Awulaelo	Ethiopia	(Biannually) Pregnancy, gestational age, delivery and stillbirth outcomes				
Kintampo	Ghana	(Biannually) pregnancy and stillbirth outcomes	Micronutrient initiatives			
Kombewa	Kenya	Demographic updates and surveillance is done twice a year (births, deaths and pregnancy outcomes)	Birth registration, verbal autopsy, migration, pregnancy surveillance and morbidity	Village team reporters		Electronic data collection with PDAs/notebook PCs and the data are stored in the MySQL database
Kyamulimbwa	Uganda	Annual census, supplemented by real-time reporting from village health workers	Pregnancy and outcomes, births and deaths	Census team, village recorders	Either the head of household or women of reproductive age	Data are collected directly onto ultra-mobile personal computers. Pregnancy registration using mobile phone app (DoForm) by village health workers
Mbita	Kenya	Every 3 months in Mbita and 4 months in Kwale	Vaccination, nutritional status of children, pregnancy surveillance (update pregnancy status, outcome, antenatal care and place of deliver), migration			Electronic data collection with PDAs and the data are stored in the MySQL database
Nairobi	Kenya	Every 4 months	Pregnancy registration, verbal autopsy pregnancy outcomes, migrations, spatial data and vaccines	Team of trained fieldworkers from the HDSS area with a minimal of O level certificate of education. VAs are conducted by team supervisors with a minimum of Bachelor’s degree in Social sciences	Household head or other adult member of household	Data were collected using paper questionnaires until June 2015. Electronic data collection since July 2015
Nanoro	Burkina Faso	Three times a year (every four months) follow-up surveys are conducted	Births, verbal autopsy migrations, pregnancies and pregnancy outcomes, vaccination, socio-economic characteristics and verbal autopsy			Data are collected on paper and entered in the Household Registration System 2
Navrongo	Ghana	(Three times a year) Pregnancy, stillbirth outcomes, birthweight and neonatal morbidity	Reproductive health, vaccination, impact assessment			
Nouna	Burkina Faso					Data are collected on pocket PCs and stored on the SQL server pro
Ouagadougou	Burkina Faso	One round every 10 months	Pregnancy registration, deaths, pregnancy outcomes, migrations, spatial data and vaccines		Adult member of the household	Data are collected on pocket PCs and stored on the MySQL server pro
Puworejo	Indonesia		Reproductive health and verbal autopsy			
Rakai	Uganda	(Started 2015) Pregnancy surveillance and stillbirth outcomes	Pregnancy and outcome surveillance, verbal autopsy, migration (of the mother), HIV status and HIV care of the mother, birth history information (including family planning use) and socio-economic status	Research assistantsN (2015	1. Migration and socio-economic status: any eligible household member 2. Verbal autopsy: any closest caregiver 3. Pregnancy outcomes, birth history, HIV status: the woman	Electronic data collection using laptops and stored in SQL database
Rufiji	Tanzania	(Three times a year) Pregnancy, stillbirth outcomes, birthweight and neonatal morbidity and maternal deaths	Pregnancy and outcome surveillance, verbal autopsy, migration and socio-economic status			Site uses OpenHDSS platform as the database since 2012
Siaya	Kenya	(Three times a year) Pregnancy outcomes, birthweight and neonatal morbidity and maternal deaths	Migration, verbal autopsy and pregnancy surveillance			Electronic data collection with PDAs/Notebook PCs using the mobile Household Registration System and stored on the MySQL database
Taabo	Côte d’Ivoire	Three times a year (every four months	Births, pregnancies (probable date of conception, gestational age, course of pregnancy), migrations, epidemiology and verbal autopsy	12 Permanent enumerators and 6 supervisors	Adult member of household	The data are entered into the Household Registration System 2 and store on the MySQL server
Vadu	India	Biannually, births, deaths, marriages, migrations and pregnancy. Every reported death is subjected to a verbal autopsy	Reproductive health, telemedicine, spatial data and verbal autopsy	12 Field research assistants	Adult member of the household who can give information	Electronic data capture on tablets using android application

The characteristics of the surveillance systems of the 31 Health and Demographic Surveillance Sites located in 13 countries for the period (2009–2014)

### Comparison to national and regional rates

While national and sub-national CRVS and HMIS systems are being strengthened, most countries with INDEPTH HDSS sites rely on five-yearly Demographic and Health Surveys and WHO-generated estimates for national mortality rates. Good quality data generated from sites can be helpful in strengthening and testing the quality of other mortality data sources. Sites with more frequent surveillance rounds, for example twice or thrice a year, and those that directed questions to women of childbearing age in the household, rather than just the household head, had SBR and NMR in a plausible range, which were also closer to regional and national estimates.

## Discussion

INDEPTH Network sites have potential for addressing key data, innovation and programme monitoring needs, especially with regard to the ENAP, the UN Strategy for Women and Children, the EPMMD strategy, key components of the SDG agenda, as well as to the emerging field of pregnancy- and childhood-related pharmacovigilance. However, we found a wide range of stillbirth and neonatal mortality rates across the different sites, suggesting huge variations in site capacities for measuring birth outcomes. The different methods for capturing pregnancy and birth outcome data across sites are likely to contribute to underreporting of events that occur around the time of birth. The findings suggest that in many sites, pregnancies and negative pregnancy outcomes are missing. These variations are likely due to limited site focus and capacity on maternal and newborn research and/or reflect difficulties in measuring these events.

Based on the international literature, the expected ratio of stillbirths to neonatal deaths is around 1. However, we found that in these settings, the median ratio of SBR to NMR was 0.8, but three sites had a ratio of SBR to NMR of ≤ 0.3, likely representing substantial underrecording of stillbirths compared to neonatal deaths (Blencowe et al. 2016). Importantly, the definition of stillbirth was consistent across sites and consistent with the World Health Organization definition for international comparison of a death at a birthweight of 1000 g or more, if the birthweight is not available, a gestational age of 28 weeks or more or a length of 35 cm or more. However, the majority of the sites rely on a woman’s recall of her last menstrual period to determine gestational age, which may not be accurate, and most stillbirths are not weighed at birth. Innovative methods are urgently needed to ensure that every pregnancy outcome is measured in terms of birthweight. Generally, stillbirths are more poorly recorded than newborn deaths. However, there may be underrecording for both stillbirths and neonatal deaths in some of the HDSS sites. The two sites with the highest ratio of stillbirths to neonatal deaths were located in South Africa, which may be plausible given the increased access to health services that are succeeding at reducing deaths among live born neonates.

With regard to neonatal mortality, sites reported rates from less than 10 per 1000 live births to 41.6 deaths per live births. Such a range is indicative of problems in data capture. Similarly, sites reporting on neonatal mortality indicated that only 1.3% of all neonatal deaths occurred on the first day, which is counter to common understanding that 75% of deaths occur in the first week, with 40 per cent in the first day after birth (Engmann et al. 2012; WHO 2011). In addition, the proportion of deaths in the first year attributed to neonatal deaths ranged from 22.6% (which likely means underreporting of neonatal events) to 100% (suggesting an absence of accurate capture of infant mortality, which is typically twice the neonatal mortality rate). We also found that the neonatal mortality rates in the HDSS were lower than the national average for the country in most sites. Several reasons could explain this, including possible underreporting by HDSS sites, differences in methods of measurement or actual lower mortality in HDSS sites due to increased services and monitoring over many years. The HDSS site populations are generally in small defined areas which are often subject to interventions or routine measurements, undertakings which can be associated with mortality reduction, and hence lower rates. However, whereas actual NMR could be lower, we do think that the quality of surveillance in HDSS sites around the time of birth is a major contributor. For instance, we found that the type of surveillance system used by sites mattered. We recommend that surveys be more frequent (e.g. 2–3 times a year) and they should target women of childbearing age themselves as the respondents, not other heads of households.

On the other hand, sites which registered pregnancies upon locating a live baby in the routine household surveillance round (“pregnancy at birth”) tended to have lower recorded mortality rates compared to those that assessed neonatal deaths by registered pregnancy. This suggests that some of the options for improving capture of events around pregnancy in HDSS sites could include shorter recall periods (more rounds), having women of childbearing age as respondents and improving pregnancy registration and surveillance for outcomes. However, it should be noted that these options are more logistically intense and expensive. In addition, by interviewing only women of childbearing age, we are likely to miss a few pregnancy outcomes in cases of a maternal death, which is itself known to be associated with the death of a baby. The finding that in some sites stillbirth and NMR were lower than national averages could suggest that either HDSS sites underestimate these events or national surveys such as DHS could be overestimating such events. National estimates—often calculated through modelling exercises based upon small samples of relatively rare events—are derived from methods that are, in general, more susceptible to bias, particularly recall bias and biases associated with the taboo of discussing bad events.

The need to improve surveillance for pregnancy and outcomes in HDSS sites has recently been emphasized (Lawn et al. 2014; Moxon et al. 2015), and our findings corroborate this recommendation. Better surveillance could play a major role in addressing the SDG measurement agenda, especially that related to the ENAP and the EPMM plans, particularly since most HDSS sites are located in countries with the highest burden of stillbirths and neonatal mortality. The presence of several HDSS sites in a country or region allows for country and regional comparisons, and the richness of high-quality HDSS data can contribute to equity analyses and information on background characteristics and risk factors contributing to mortality. The prospective nature of HDSS data and availability of cause of death data also allow for better programme targeting over time, as the mortality share of different diseases can change. Improving pregnancy and outcome surveillance will also provide INDEPTH with a platform to monitor scale-up and safety of new and old interventions such as immunization and medicines taken during pregnancy, and any unintended consequences such as birth defects. As such, HDSS data can be a powerful precision public health tool to inform programming, advocacy and testing new innovations.

For HDSS sites to achieve their full potential, however, data quality needs to be prioritized throughout the network. There is a need to standardize pregnancy and outcome data collection tools, indicator definition and analyses. This includes causes of death which were not included in this analysis but merit discussion. Current analyses of verbal autopsy data in INDEPTH HDSS use ICD-10 rules and do not group deaths into programmatically relevant categories as has been done by the Child Health Epidemiology Reference Group and others (Lawn et al. 2006, 2014; Liu et al. 2014; Lozano et al. 2012; Wang et al. 2014). For instance, several studies have shown the growing importance of prematurity as a cause of death and disability and it is now the leading cause of under-five deaths worldwide (Liu et al. 2014). However, in current analyses of cause of death data in HDSS sites, prematurity does not feature highly (Streatfield et al. 2014). The increasing recognition of the HDSS potential has seen them to become the leading centres for research on morbidity surveillance such as the CHESS/CHAMPS studies and the minimally invasive autopsy (MIA), which aim to generate more precise causes of morbidity and mortality.

Research implications include further work needed on pregnancy validation, stillbirth and neonatal measurement, and cause of death coding. Fortunately, through work in the ENAP measurement agenda, work has started within INDEPTH to validate current surveillance systems and ENAP indicators, improve surveillance of pregnancy and outcomes and develop more accurate neonatal and verbal autopsy cause of death algorithms. This work will lead to improved measurement and will also inform other survey systems such as DHS and the Multiple Indicator Cluster Surveys (MICS). In broader terms, the INDEPTH Network should strengthen its systems to be able to validate its potential to monitor almost all indicators for SDGs.

A major strength of this study is that it includes data from 31 sites, representing a wide range of settings and surveillance systems. However, the study has some important limitations. The data are reported here as presented to the MNCH_WG, although several efforts were made to validate the data by having meetings and returning queries to sites. Furthermore, despite attempts to standardize definitions, some outcome variables could have varied across sites. The national and regional comparisons should be used with caution because the methods for estimating the national rates from global estimates and/or demographic and health surveys are not comparable to the timing or methodology used in the HDSS. Sub-national mortality data for the same time period are not available for most of these sites, highlighting the importance of the HDSS sites in contributing to national data. Given the background characteristics of households and individuals available in the HDSS databases, there is a potential to further explore these explanatory variables in the context of birth outcomes and mortality rates. In addition, future analyses could describe how different factors across sites, such as length of operation, incentives and surveillance timing, contribute to trends in data capture, particularly for pregnancy outcomes.

### Conclusion

Greater understanding of the high risk of death in the days around and immediately after birth can help HDSS researchers and funders prioritize learning around urgently needed interventions, with information shared with policy makers and healthcare providers for wider uptake. The rich information available across INDEPTH sites can help to contribute towards bridging knowledge and action gaps around the time of birth and the subsequent weeks. INDEPTH has an important role to play in ENAP and the broader global maternal, newborn, child health and adolescent agenda. It is also a critical platform in the testing of new medicines and vaccines, interventions and packages and the ability to provide long-term follow-up, especially for preterm and birth asphyxia outcomes.

Nonetheless, for this potential to be achieved, there is need for INDEPTH Network sites to improve data capture and intentionally focus on metrics for pregnancy and the time of birth, given the massive burden of deaths and morbidity. The underreporting of pregnancies and birth outcomes is a major issue which needs to be addressed in order to achieve the full potential of the network. While all HDSS centres track pregnancies and their outcomes, extra attention to ensuring complete collection as well as standardized data collection systems for birth outcomes is required, in order to advance the work around this crucial time period to prevent maternal deaths, stillbirths and neonatal deaths. To bridge these gaps, studies are underway to identify the best methods for capturing pregnancies and their outcomes. However, if these sites are to achieve their full potential in the SDG era, they will require strategic funding, innovations around measurement and capacity building and partnerships.

## Electronic supplementary material

Below is the link to the electronic supplementary material.

Supplementary material 1 (DOCX 44 kb)

Supplementary material 2 (DOCX 30 kb)
