# Supplementary material for: Status of birth and pregnancy outcome capture in Health Demographic Surveillance Sites in 13 countries
Source: Int J Public Health. 2019 Jun 26;64(6):909–20. doi: 10.1007/s00038-019-01241-0 (PMC6614155; doi:10.1007/s00038-019-01241-0)
Supplement: Supplementary file 1 — Supplementary material 1 (DOCX 44 kb) [file 38_2019_1241_MOESM1_ESM.docx]

**Table 2: Health Demographic Surveillance Site characteristics and surveillance system details**

| **HDSS name** | **Country and Region** | **HDSS**  **Population** | **Data**  **year** | **HDSS**  **Area (km2)** | **Brief  description #** | **HDSS**  **start year** | **Submitted population and birth outcome data for this analysis (Y/N)** |
| --- | --- | --- | --- | --- | --- | --- | --- |
| Africa Centre | South Africa  *Sub-Saharan Africa* | 88977 | 2011 | 438 | Predominantly rural and is located in the Umkanyakude district of KwaZulu-Natal | 2000 | Yes |
| Agincourt | South Africa  *Sub-Saharan Africa* | 90168 | 2011 | 420 | Located in the rural northeast South Africa. It is comprised of 27 villages | 1992 | Yes |
| AHRI | South Africa  *Sub-Saharan Africa* | 168000 |  | 440 | Located in the southern part of Umkhanyakude district KwaZulu-Natal (Mpukunyoni) | 2000 | No |
| Ballabgarh | India  *Central Asia and Southern Asia* | 92072 | 2012 | 301 | Located in district Faridabad in the province of Haryana in northern part of India. It consists of rural population residing in 28 villages | 1961 | Yes |
| Bandafassi | Senegal  *Sub-Saharan Africa* | 13373 |  |  | Located southeastern Senegal. It is comprised of 42 villages which are largely rural | 1970 | No |
| Bandarban | Bangladesh  *Central Asia and Southern Asia* | 19403 |  | 4479 | Located in the southeast of Bangladesh in 2 rural unions of Kuhalong and Rajbila Bandarban district. It is predominantly a rural site |  | No |
| Bandim | Guinea-Bissau  *Sub-Saharan Africa* | 109576 | 2014 |  | The site covers rural and urban areas with an approximate population of 90,000 people in each area. | 1978 | Yes |
| Birbhum | India  *Central Asia and Southern Asia* | 59395 |  |  | Located in Birbhum district of west Bengal, India. It covers 351 villages in four administrative blocks. It is predominantly a rural site | 2008 | No |
| Butajira | Ethiopia  *Sub-Saharan Africa* | 78694 | 2014 |  | Located in one of the most densely populated parts of Ethiopia, Meskan, Mareko and Silti districts. It covers 10 kebeles/villages (1 urban and 9 rural) | 1986 | Yes |
| Chakaria | Bangladesh  *Central Asia and Southern Asia* | 120000 |  | 288 | Located southeastern coast of the Bay of Bengal in the district of Chakaria Upazila. It is a rural HDSS site | 1999 | No |
| Chókwè | Mozambique  *Sub-Saharan Africa* | 99834 |  |  | Located in the southern part of Mozambique, 230 km north of the Maputo (capital city) in the rural town of Chókwè district, Gaza province | 2010 | No |
| Cross River | Nigeria  *Sub-Saharan Africa* | 31124 |  |  | Located in Cross River State. It covers both rural and urban areas. The rural site is located in Ikot Nakanda communities, Akpabuyo, and Cross River state and the urban site is located in Ikot Ishie, Ikot Ansa communities, Calabar municipality and Cross River state | 2010 | No |
| Dabat | Ethiopia  *Sub-Saharan Africa* | 69468 | 2014 |  | Located approximately 821 km northwest of Addis Ababa and 75km north of Gondor town. The site covers 13 selected kebeles(9 are rural and 3 are urban) | 1996 | Yes |
| Dikgale | South Africa  *Sub-Saharan Africa* | 35000 |  |  | Located about 40km northeast of Polokwane, Limpompo Province, and Northeastern South Africa. | 1996 | No |
| Dodalab | Vietnam  *Eastern Asia and Southeastern Asia* | 40598 |  |  | Located in Dong Da district of Hanoi city, the capital of Vietnam. It covers 3 of the 21 communes in Honai. It is an urban site | 2007 | No |
| Dodowa | Ghana  *Sub-Saharan Africa* | 117341 | 2013 | 1528 | Dodowa Health Research Centre is placed in Dodowa, the district capital of the Danmbe West district. The Dangme West district is divided into four sub-districts (Dodowa, Ningo, Osudoku and Prampram) and has seven area councils (Ayikuma, Asutsuare, Dodowa, Dawa, Ningo, Osuwem and Prampram). | 1990 | Yes |
| Farfenni | Gambia  *Sub-Saharan Africa* | 47331 | 2013 |  | Located 170km from the coast in rural Gambia, It covers 40 villages | 1981 | Yes |
| Filabavi | Vietnam  *Eastern Asia and Southeastern Asia* | 51797 |  |  | Located in Bavi, one of the 29 districts in Honai |  | No |
| Gilgel Gibe | Ethiopia  *Sub-Saharan Africa* | 63081 | 2014 | 202 | Gilgel Gibe is composed of 8 rural kebeles and 3 towns from four districts surrounding the Gilgel Gibe hydroelectric dam in the South West of Ethiopia |  | Yes |
| Ifakara | Tanzania  *Sub-Saharan Africa* | 133588 | 2011 |  | Located in southern Tanzania in parts of two districts; Kilombero and Ulanga. Both in Morogoro region. The site covers 25 villages in rural area of Ulanga and Kilombero | 1997 | Yes |
| Iganga-Mayuge | Uganda  *Sub-Saharan Africa* | 82336 | 2011 | 155 | Predominantly peri-urban with 80% rural with 65 villages in two districts Iganga and Mayuge. Located in Eastern Uganda, approximately 120km along the Kenya-Uganda highway from capital city Kampala. | 2004 | Yes |
| Karonga | Malawi  *Sub-Saharan Africa* | 35398 | 2011 | 135 | Located in the rural area of Karonga district in the north of the country near the lake Malawi | 2002 | Yes |
| Kaya | Burkina Faso  *Sub-Saharan Africa* | 60287 | 2011 |  | The Kaya health district in the North Central region is 70% rural and 30% urban. It covers 18 villages in the rural and a town in the urban | 2007 | Yes |
| Kersa | Ethiopia  *Sub-Saharan Africa* | 62469 | 2014 | 249 | Located in Kersa district of eastern Hararege zone of Oromia region, eastern Ethiopia. runs in 12 representative Kebeles out of the 38 in Kersa districts. | 2007 | Yes |
| Kilifi | Kenya  *Sub-Saharan Africa* | 268783 | 2012 | 891 | Located on the Indian Ocean coast of Kenya. Mainly rural with two urban centres | 2000 | Yes |
| Kilite- Awlaelo | Ethiopia  *Sub-Saharan Africa* | 64575 | 2012/13 |  | Located 802km north of Addis Ababa in Tigray one of the nine administrative regions of Ethiopia. The Kiltie Awlaelo HDSS includes 10 kebeles (districts) | 2009 | Yes |
| Kintampo | Ghana  *Sub-Saharan Africa* | 150407 | 2013 | 7162 | Located in the middle belt of Ghana in the Brong Ahafo region. It is predominantly rural and primary occupation is subsistence agriculture | 1994 | Yes |
| Kombewa | Kenya  *Sub-Saharan Africa* | 137987 | 2012 | 369 | HDSS grew out of the Kombewa Clinial Research center. It is located in the rural part of Kisumu county | 2007 | Yes |
| Kyamulimbwa | Uganda  *Sub-Saharan Africa* | 20630 | 2014 | ?? | Located in Kalungu district (former Masaka district), central Uganda. It is predominantly a rural site with 25 adjacent villages, including a population of about 22,000 people of all ages | 1989 | Yes |
| Manhica | Mozambique  *Sub-Saharan Africa* | 89617 |  | 2.373 | Located in Manhic,a district, 80km north of Maputo (capital city) | 1996 | No |
| Matlab | Bangladesh  *Central Asia and Southern Asia* | 225000 |  | 184 | Located in Matlab Upazila sub-district under the Chandpur district Bangladest. Located 55km southeast of Dhaka (Capital city) | 1966 | No |
| Mbita | Kenya  *Sub-Saharan Africa* | 58214 | 2012 | 163 | Located on the shores of Lake Victoria in Kenya. It is predominantly in the rural area. The population lives on subsistence farming, small scale businesses, fishing and rearing of animals | 2006 | Yes |
| Mlomp | Senegal  Sub- Saharan Africa | 8200 |  | 70 | Located in thr Ziguinchor region, department of Oussouye, southwestern Senegal covering 11 villages (each composed of a circle of 3-km diameter) | 1985 | No |
| Nairobi | Kenya  *Sub-Saharan Africa* | 83531 | 2011 |  | Urban based HDSS located in two slums in Nairobi (Korogocho and Viwandani) | 2002 | Yes |
| Nanoro | Burkina Faso  *Sub-Saharan Africa* | 61927 | 2011 | 594 | The HDSS covers 24 villages belonging to two departments (Nanoro and Soaw), located in the Boulkiemdé province. | 2009 | Yes |
| Navrongo | Ghana  *Sub-Saharan Africa* | 161415 | 2014 | 1675 | Located in the Kassena-Nankana district of upper east region of Ghana. The surveillance area has been divided into five zones with 247 clusters | 1993 | Yes |
| Niakhar | Senegal  *Sub-Saharan Africa* | 43000 |  | 203 | Located in Niakhar rural Senegal, approximately 135km east of Dakar (capital city). Between 1962-69 it covered 65 villages, this was reduced to 8 villages between 1969-83. Later 22 villages were added to the 8 villages making it 30 villages under surveillance. | 1962 | No |
| Nouna | Burkina Faso  *Sub-Saharan Africa* | 93000 | 2011 | 1775 | Located within the Nouna health district catchment area in northwest Burkina Faso. It covers 58 villages and Nouna town located 300km from the capital city. | 1992 | Yes |
| Ouagadougou | Burkina Faso  *Sub-Saharan Africa* | 89498 | 2011 |  | Located in five neighbourhoods at the northern periphery of Ouagadougou. Burkina Faso | 2008 | Yes |
| PiH | Papua New Guinea  *Oceania* | 56000 |  |  | Located in Alotau Goroka, Madang, Maprik, Port Moresby, Wewak, Hides-Tari and Hiri West. | 1968 | No |
| Puworejo | Indonesia  *Eastern Asia and South-eastern Asia* | 52767 | 2010 | 1034 | Located in the district of Puworejo, province of central Java. It is comprised of 16 sub-districts, 494 villages | 1994 | Yes |
| Rakai | Uganda  *Sub-Saharan Africa* | 50109 | 2011/13 |  | The HDSS covers 11 super clusters with 80% of the population in rural communities | 1994 | Yes |
| Rufigi | Tanzania  *Sub-Saharan Africa* | 102883 | 2012 | 1813 | Located in Rufigi district. It is comprised of 38 villages which are largely rural | 1998 | Yes |
| Sapone | Burkina Faso  *Sub-Saharan Africa* | 86069 |  | 1700 | Located in central Burkina Faso, but south of the capital city Ouagadougou. It is predominantly rural |  | No |
| SEACO | Malaysia  *Eastern Asia and Southeastern Asia* | 40000 |  | 1250 | Located in Segamat, the northernmost district in the southern peninsular state of Johor. It covers 11 sub-districts of Segmat | 2011 | No |
| Siaya | Kenya  *Sub-Saharan Africa* | 234648 | 2012 |  | The HDSS is located in Siaya county, lying northeast of Lake Victoria. Has 36 health facilities within its catchment area | 2003 | Yes |
| Taabo | Côte d'Ivoire  *Sub-Saharan Africa* | 41351 | 2012 |  | Located 150 km north-west of Abidjan. HDSS consists of a small town, 13 villages and over 100 hamlets | 2008 | Yes |
| Vadu | India  *Central Asia and Southern Asia* | 115781 | 2012 | 232 | Located 30km to the northeast of Pune city covering 22 villages | 2002 | Yes |

*Forty eight Health and Demographic Surveillance Sites that were contact and profiles reviewed during the periods 2009 - 2014*

***# - Source:*** The International Network for the Demographic Evaluation of Populations and their Health *(INDEPTH)* *NETWORK member HDSS profiles*[***^23^***](#_ENREF_23)
