# Supplementary material for: Status of birth and pregnancy outcome capture in Health Demographic Surveillance Sites in 13 countries
Source: Int J Public Health. 2019 Jun 26;64(6):909–20. doi: 10.1007/s00038-019-01241-0 (PMC6614155; doi:10.1007/s00038-019-01241-0)
Supplement: Supplementary file 2 — Supplementary material 2 (DOCX 30 kb) [file 38_2019_1241_MOESM2_ESM.docx]

**Table 3: Stillbirths and neonatal deaths across sites (2009-2014 average)**

| **HDSS** | **Population** | **Live births** | **Stillbirth**  **rate*** | **Early NMR** | **NMR** | **IMR** | **SBR:NMR**  **Ratio** | **Proportion of NMR to IMR** | **National**  **SBR (2015)#** | **National**  **NMR (2015)#** |
| --- | --- | --- | --- | --- | --- | --- | --- | --- | --- | --- |
| Agincourt  *South Africa*  *Sub-Saharan Africa* | 90168 | 6429 | 17.9 | 7.3 | 11.0 | 32.7 | 1.6 | 0.3 | 17.4 | 11.0 |
| Ballagbarh  *India*  *Central Asia and Southern Asia* | 92072 | 11580 | 11.0 | 20.8 | 28.3 | 50.9 | 0.4 | 0.6 | 23.0 | 27.7 |
| Bandim  *Guinea-Bissau*  *Sub-Saharan Africa* | 109576 | 12402 | 42.6 | 29.2 | 35.1 | 56.2 | 1.2 | 0.6 | 36.7 | 39.7 |
| Butajira  *Ethiopia*  *Sub-Saharan Africa* | 78694 | 1065 | 17.5 | 17.8 | 23.5 | 31.9 | 0.7 | 0.7 | 29.7 | 27.7 |
| Dabat  *Ethiopia*  *Sub-Saharan Africa* | 69468 | 1267 | 17.8 | 16.1 | 28.0 | 27.7 | 0.6 | 1.0 | 29.7 | 27.7 |
| Farfenni  *Gambia*  *Sub-Saharan Africa* | 47331 | 1885 | 23.8 | 12.7 | 14.9 | 30.8 | 1.6 | 0.6 | 23.9 | 29.9 |
| Gigel Gibe*  *Ethiopia*  *Sub-Saharan Africa* | 63081 | 1993 | 17.0 | 27.8 | 34.0 | 59.1 | 0.5 | 0.6 | 29.7 | 27.7 |
| Ifakara  *Tanzania*  *Sub-Saharan Africa* | 133588 | 11447 | 8.2 | 24.6 | 28.1 | 49.7 | 0.3 | 0.6 | 22.4 | 18.8 |
| Iganga-Mayuge  *Uganda*  *Sub-Saharan Africa* | 82336 | 8850 | 9.1 | 24.3 | 27.9 | 53.6 | 0.3 | 0.5 | 21.0 | 18.7 |
| Karonga  *Malawi*  *Sub-Saharan Africa* | 35398 | 5277 | 18.2 | 11.7 | 19.8 | 26.9 | 0.9 | 0.7 | 21.8 | 21.8 |
| Kaya  *Burkina Faso*  *Sub-Saharan Africa* | 60287 | 2553 | 17.3 | 0.0 | 12.1 | 40.7 | 1.4 | 0.3 | 21.2 | 26.7 |
| Kersa  *Ethiopia*  *Sub-Saharan Africa* | 62469 | 2016 | 12.0 | 12.9 | 25.8 | 50.1 | 0.5 | 0.5 | 29.7 | 27.7 |
| Kilifi  *Kenya*  *Sub-Saharan Africa* | 268783 | 8152 | 11.3 | 11.9 | 14.3 | 25.0 | 0.8 | 0.6 | 22.5 | 22.2 |
| Kilite-Awlaelo  *Ethiopia*  *Sub-Saharan Africa* | 64575 | 1419 | 13.9 | 19.7 | 24.0 | 31.7 | 0.6 | 0.8 | 29.7 | 27.7 |
| Kintampo  *Ghana*  *Sub-Saharan Africa* | 150407 | 4787 | 20.9 | 18.2 | 20.5 | 33.8 | 1.0 | 0.6 | 22.7 | 28.3 |
| Kyamulimbwa  *Uganda*  *Sub-Saharan Africa* | 20630 | 381 | 18.4 | 37.4 | 41.6 | 49.9 | 0.4 | 0.8 | 21.0 | 18.7 |
| Mbita  *Kenya*  *Sub-Saharan Africa* | 58214 | 4539 | 11.1 | 8.2 | 11.5 | 50.9 | 1.0 | 0.2 | 22.5 | 22.2 |
| Nairobi  *Kenya*  *Sub-Saharan Africa* | 83531 | 2148 | 12.9 | 15.4 | 24.2 | 60.5 | 0.5 | 0.4 | 22.5 | 22.2 |
| Nanoro  *Burkina Faso*  *Sub-Saharan Africa* | 61927 | 5519 | 7.0 | 8.7 | 12.9 | 36.6 | 0.5 | 0.4 | 21.2 | 26.7 |
| Navrongo  *Ghana*  *Sub-Saharan Africa* | 161415 | 4130 | 11.5 | 9.4 | 12.3 | 26.4 | 0.9 | 0.5 | 22.7 | 28.3 |
| Puworejo  *Indonesia*  *Eastern Asia and South-eastern Asia* | 52767 | 199 | 15.2 | 8.1 | 12.3 | 20.5 | 1.2 | 0.6 | 13.2 | 13.5 |
| Rufigi  *Tanzania*  *Sub-Saharan Africa* | 102883 | 10384 | 17.0 | 18.1 | 22.2 | 40.7 | 0.8 | 0.5 | 22.4 | 18.8 |
| Siaya  *Kenya*  *Sub-Saharan Africa* | 234648 | 6666 |  | 10.6 | 14.8 | 63.8 |  | 0.2 | 22.5 | 22.2 |
| Taabo  *Côte d'Ivoire*  *Sub-Saharan Africa* | 41351 | 5317 | 19.0 | 9.2 | 11.7 | 44.4 | 1.6 | 0.3 | 26.7 | 37.9 |

Stillbirth and neonatal deaths of the surveillance systems of 24 Health and Demographic Surveillance Sites located in 13 countries for the period (2009 – 2014)

**Definition of stillbirth at Gilgel Gibe: Babies born per year with no signs of life after 27 completed weeks of gestation*

**# *Source: Health Newborn Network (***[***https://www.healthynewbornnetwork.org/numbers/***](https://www.healthynewbornnetwork.org/numbers/)***)***

***Note: Data from seven sites is not shown because they did not provide any data on the neonatal deaths or the neonatal mortality rates computed were less than 10 deaths per 1000 live births***
